# Hip Fracture Surgery during COVID-19 Pandemic

**DOI:** 10.5704/MOJ.2203.026

**Published:** 2022-03

**Authors:** R Mungmunpuntipantip, V Wiwanitkit

**Affiliations:** 1 Bangkok, Thailand; 2 Department of Community Medicine, Dr. D. Y. Patil Medical College Hospital and Research Centre, Pune, India

Dear editor,

We would like to share ideas on the publication “Mortality Following Hip Fracture Surgery During COVID-19 Pandemic Compared to Pre-COVID-19 Period: A Case Matched Cohort Series1.” De *et al* concluded that “There is strong association between 30-day mortality and the designated theatre (Clean/COVID) … symptomatic or COVID-19 positive patients“^1^. There are several reports on the effect of COVID-19 on the clinical service. The effect on increased mortality is similar to a recent report from United Kingdom^2^. For management of a patient with hip fracture, there might be a delay, which can cause a poor clinical outcome.

In the present study, whether there is an effect of a delay or not is an interesting point for further study. Regarding the present report on mortality, there are many confounding factors. In a different COVID-19 case, severity might be different.

In a recent report, there is an association between the period of hospitalisation, which might be indirectly related to COVID-19 severity, and post-operative mortality rate^3^. Subgroup analysis, based on COVID-19 severity classification, is recommended. 
